# Characterization of near-complete hepatitis E virus genomes of genotype 1e and 4b detected from humans in Cameroon

**DOI:** 10.1128/mra.00767-25

**Published:** 2025-11-10

**Authors:** Abdou Fatawou Modiyinji, Pierre Cappy, Arnaud Ly, Aristide Mounchili-Njifon, Moise Henri Yifomnjou Moumbeket, Huguette Tchetgna Simo, Abanda Njei Ngu, Richard Njouom

**Affiliations:** 1 Centre Pasteur du Cameroun, Service de virologie 567903, Yaoundé, Cameroon; 2 Plateforme GenoBioMICS, Laboratoire de virologie, Hôpitaux Universitaires Henri Mondor 378967, Créteil, France; Katholieke Universiteit Leuven, Leuven, Belgium

**Keywords:** hepatitis E virus, genome, genotype, Cameroon

## Abstract

Hepatitis E virus (HEV) is an important public health concern, especially in developing countries. Complete genomes of HEV strains circulating in Cameroon are not available. Here, we report five nearly complete strains of HEV in Cameroon. These strains share a high identity with human African and swine Asian isolates.

## ANNOUNCEMENT

Hepatitis E virus (HEV) is probably the most common cause of acute hepatitis in humans worldwide (1). HEV is a single-stranded, positive-sense RNA virus with a genome size of approximately 7.2 kb (2). Human HEV belongs to the genus *Paslahepevirus*, subfamily *Orthohepevirinae,* and the family *Hepeviridae*. The members of species *Paslahepevirus balayani* have been classified into eight genotypes (HEV-1 to 8), of which five are well recognized as human pathogens (HEV-1 to 4 and HEV-7) (2). HEV-1 and 2 are transmitted by the fecal-oral route and are responsible for significant waterborne outbreaks in developing countries (2). HEV-3 and 4 are mainly transmitted zoonotically and are responsible for sporadic infections in developed countries (2). HEV-7 was associated with chronic infection in a liver transplant recipient from the Middle East (3). Some of these eight genotypes can be divided into subtypes (4).

It has been reported that HEV-1e was responsible for a large outbreak in sub-Saharan Africa (5). However, prior to our recent study (6), HEV-4b strain has never been reported in Africa. To date, no complete HEV genome is available from Cameroon. We report the near full-length genome sequences of an HEV-1e and HEV-4b from Cameroon. The viruses were detected in our previous study from icteric patients suspected of having yellow fever in two regions of Cameroon (6). Plasma samples from these patients were negative for yellow fever virus, and molecular tests using partial sequencing of the ORF2 region identified HEV-1e and 4b. We selected five plasma samples collected in 2022 for complete genome characterization using metagenomics followed by hybrid–capture enrichment, as previously described (7). Briefly, total nucleic acid extractions were performed using the QIAsymphony DSP DNA Midi kit (Qiagen, Hilden, Germany), according to the manufacturer’s instructions. From the extracted nucleic acids, we performed cDNA synthesis, tagmentation, PCR indexing, purification, and normalization of the libraries produced. Then, we proceeded to the hybridization of biotinylated capture probes and to the capture of probe-library hybrids using magnetic beads coupled with streptavidin. After washing and elution, we proceeded to the re-amplification of the libraries and the clean-up of the final library. Mixed DNA/RNA libraries were sequenced on a NovaSeq 6000 sequencer (Illumina). The raw data were demultiplexed using BCLConvert on a Dragen server, and Fastq files were then analyzed on the BaseSpace cloud (Illumina), using Dragen Microbial Enrichment Plus software, to obtain HEV consensus sequences.

**Fig 1:**
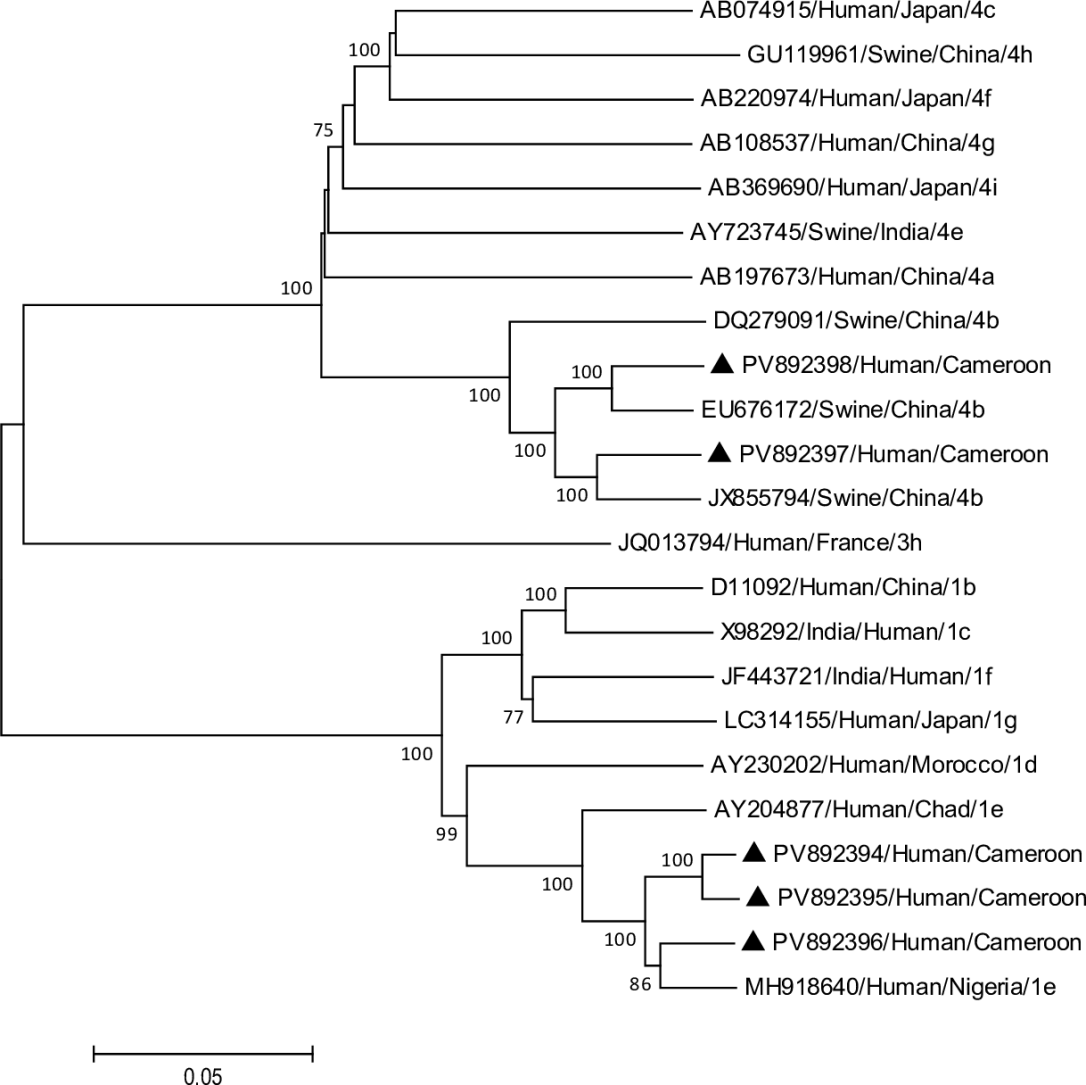
Phylogenetic tree based on near-complete genomes of HEV strains available in GenBank. The accession number, host, country of origin, and genotype/subtype are shown for each HEV strain used in the phylogenetic analysis. The strains identified in this study are indicated by ▲. Numbers along branches indicate bootstrap values. Only bootstrap values >70% are presented. Phylogenetic analysis was performed using the maximum likelihood method and the Kimura two-parameter model, using MEGA software (version 7).

A total of five near-complete sequences were obtained. Three strains form a cluster with African HEV-1e strains (Fig. 1). These HEV-1e strains shared the highest identity (>95%) with the NG/17-0503 strain from Nigeria. Our strains have been identified in the Far North region of Cameroon, bordering Nigeria. This observation strongly suggests cross-border circulation of this virus. Two strains shared the highest identity (>95%) with the HEV-4b strains identified in swines in China (Fig. 1). This observation reinforces the hypothesis of zoonotic transmission of HEV in Cameroon (Table 1).

**TABLE 1:** Characteristic of the 5 HEV genomes identified

Accession number	Year of collection	Total reads	Aligned reads	G + C content	Closest GenBank hit	% similarity	Source country/year of sampling	SRA_ Accession
PV892394	2022	46,891,625	16,412	57.8%	MH918640	96.33%	Nigeria/2017	ERS26973331
PV892395	2022	3,072,858	108,048	57.7%	MH918640	96.26%	Nigeria/2017	ERS26973332
PV892396	2022	22,784,589	106,215	57.9%	MH918640	96.63%	Nigeria/2017	ERS26973335
PV892397	2022	55,185,294	699,296	55.2%	JX855794	95.64%	China/2011	ERS26973334
PV892398	2022	47,531,570	1,548	55.5%	EU676172	95.94%	China/2008	ERS26973333

In conclusion, these five near-complete HEV genomes from Cameroon will be an important resource for future epidemiological research in Africa.

## Data Availability

The near-complete genome sequences have been deposited in the GenBank database under the accession no. PV892394, PV892395, PV892396, PV892397, and PV892398. The raw NGS reads have been published in the European Nucleotide Archive (BioSamples) under the SRA Accession no. ERS26973331, ERS26973332, ERS26973333, ERS26973334, and ERS26973335.
